# P-253. Impact of Restroom Disinfecting/Cleaning Frequency and Product Selection on Risk of Infection

**DOI:** 10.1093/ofid/ofae631.457

**Published:** 2025-01-29

**Authors:** Charles P Gerba

**Affiliations:** University of Arizona, Tucson, Arizona

## Abstract

**Background:**

Restrooms are associated with transmission of bacterial and viral illnesses. Disinfecting pathogen-contaminated surfaces represents an intervention for reducing transmission risk. This study assessed whether cleaning/disinfecting frequency affects bacterial pathogen contamination in American patient rooms, long term care facilities and households with ≥ four occupants.

**Methods:**

During the control phase, toilet surfaces (toilet bowl, water, and rim) were cleaned using a 9.5% w/w hydrochloric acid product only. During the intervention phase, multiple restroom surfaces (vanity/sink, toilet surfaces, floor, doorknob including toilet bowl) were cleaned/disinfected with disinfecting/cleaning products selected for the specific surfaces. Surface samples were collected and tested for heterotrophic and coliform bacteria after 1, 2, 3, or 7 days, with sampling being randomized during the week. The ratio of E. coli to norovirus in feces from an infected person was then used to perform a quantitative microbial risk assessment.

**Results:**

The greatest numbers of bacteria were recovered on surfaces after three days. The largest reduction in bacterial recovery occurred during a three-day (∼twice-a-week) cleaning cycle, resulting in statistically significant reductions in recovery of heterotrophic bacteria (*P* = 0.00898) and *Escherichia coli* (*P* = 0.01).

**Conclusion:**

Quantitative microbial risk assessments of commonly touched surfaces suggests that risk of infection by norovirus (highly contagious, excreted in high numbers, low human infectious dose) may be reduced by 91.5% using a three-day (twice-a-week) restroom cleaning/disinfection cycle.

**Disclosures:**

**Charles P. Gerba, PhD**, Rickitt Benckiser: Grant/Research Support

